# CLIMATE CHANGE AND AGING: FROM VULNERABILITY TO ACTION

**DOI:** 10.1093/geroni/igad104.0497

**Published:** 2023-12-21

**Authors:** Liat Ayalon, Karl Pillemer

## Abstract

Older people are particularly susceptible to extreme climate change events. This is attributable to physiological susceptibility, but also to factors such as social isolation, digital exclusion, ageism, and living arrangement. Unfortunately, older people are often not considered in mitigation and adaptation climate change policies contributing to their heightened susceptibility in the face of extreme climate events. Compounding this challenge, it is younger people who are seen as the face of the climate change movement, at times, blaming older people for their (in)action, which has contributed to the current climate situation. Nevertheless, research has shown that older people engage in pro-environmental behaviors and can highly benefit from taking part in climate change activism. The present symposium aims to tackle this topic by discussing the challenges pertaining to older populations occasioned by climate change, and how older populations can engage in being part of mitigation solutions.. The first presentation by Carr and colleagues addresses global population aging and heat exposure with a particular focus on implications for wellbeing and social policy. Delving into the country level, Cherry and colleagues discuss findings concerning long term mental health effects of exposure to a single versus two extreme weather events. Moving to possibly mitigation and adaptation measures, Nikitin and Rupprecht address opportunities associated with exposure to nature, whereas Kohon and colleagues discuss the role that aging and old age capture in climate change policies. The symposium concludes with a theoretical conceptualization by Diehl which reframes aging in the context of climate change. This is a Climate Change and Aging Interest Group Sponsored Symposium.

